# Proximal tibial reconstruction with auto transplantation of the fibular growth plate: two case reports, describing the surgical technique

**DOI:** 10.1186/s40064-016-2042-7

**Published:** 2016-04-20

**Authors:** Pedro Péricles Ribeiro Baptista, Cassiano Leão Bannwart, Felipe Augusto Ribeiro Batista, Davi Gabriel Bellan

**Affiliations:** Orthopedic Division, Faculty of Medical Sciences, Santa Casa de São Paulo, São Paulo, Brazil; Hand Surgery Division, Faculty of Medical Sciences, Santa Casa de São Paulo, São Paulo, Brazil; Oncology Orthopedic Division, Federal University of São Paulo – UNIFESP, São Paulo, Brazil; R. General Jardim, 846 conjunto 41, CEP 01223010 São Paulo, SP Brazil

**Keywords:** Growth, Sliding, Device, Fibula, Physis, Fixation, Sarcoma, Tibialization, Transplant

## Abstract

**Introduction:**

Tumors of the proximal tibia, in children, can affect the growth plate and pose a challenge to further reconstruction of the bone defects resulting from tumor resection. Reconstruction methods do not always compensate the potential for bone growth in this segment. We present a new surgical technique of bone reconstruction, based on the transposition of the ipsilateral fibula with its growth plate and the use of an internal sliding fixation device, without need for microsurgical technique.

**Case description:**

We report two patients with osteosarcoma of the proximal tibia affecting the growth cartilage who were treated with the new technique.

**Discussion and Evaluation:**

In both cases, bone healing, hypertrophy and longitudinal growth of the transposed fibula were documented.

**Conclusions:**

This new technique preserves the blood supply of the auto-transplanted bone segment, maintaining physeal growth potential, with no need for microsurgery. The implant allows longitudinal bone growth, which was radiographically confirmed.

**Level of evidence:**

Case report, Level IV.

**Electronic supplementary material:**

The online version of this article (doi:10.1186/s40064-016-2042-7) contains supplementary material, which is available to authorized users.

## Background

**Fig. 1:**
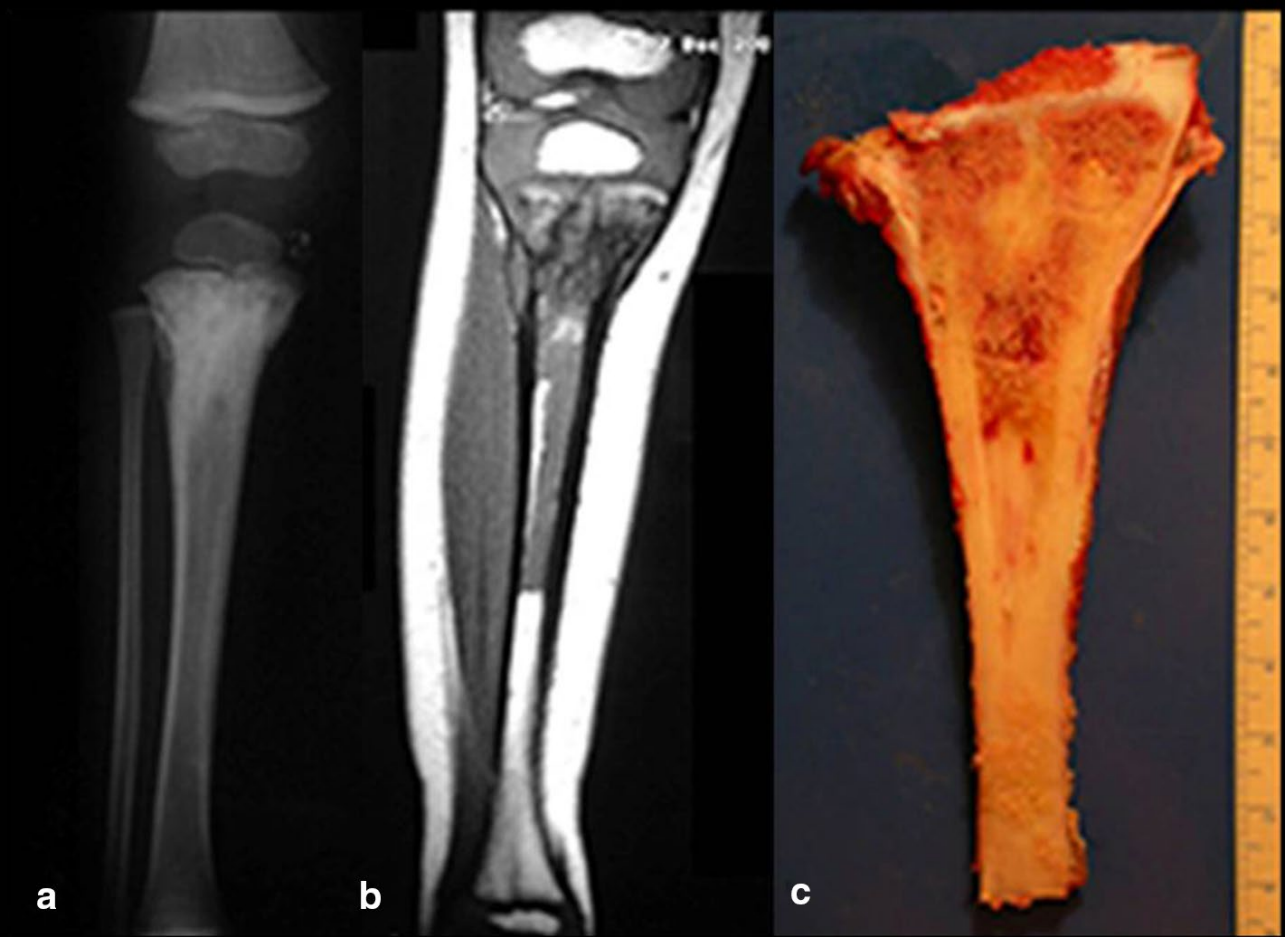
**a** Radiography of the tibia showing a proximal metaphyseal bone tumor. **b** MRI image showing no compromise of the proximal epiphysis by the tumor. **c** Resected specimen

In the skeletally immature population, the proximal tibia hosts a growth plate that accounts for nearly 30 % of the final limb length in adulthood (Digby 1916). This is the second most frequent location of primary bone tumors, with the first being the distal femur (Mercuri et al. 1991). Tumors that develop in the proximal tibia before skeletal maturity can affect the growth plate and lead to discrepancies in the final length of the lower limbs (Fig. 1).

In young patients, reconstruction of bone defects resulting from tumor resection of this segment with traditional methods may show poor results after skeletal growth (Boyer et al. 1994). Some of this current methods include the replacement of the bone segment by megaprothesis, callotasis and the use of bone autograft or allograft (Saghieg et al. 2010). None of these techniques can replace the injured growth plate. Although callotasis allows bone lengthening, it may be impractical due to the need of multiple interventions for limb equalization in young children and the prolonged use of an external fixator device, which may favour infection in immunosuppressed oncological patients.

The advance of vascularized fibula autograft by microsurgical technique has allowed its use with the functioning physis in distant locations from its anatomical site (Agiza 1981; Langenskiöld 1983; Taylor et al. 1975). This has enabled the reconstruction of bone defects while keeping its growth potential (Pho et al. 1988). This technique, however, is costly and has potential complications.

In this study we describe a new surgical technique for reconstruction of bone lesions that compromise the proximal tibia and its growth plate in children and report two cases successfully treated with this technique. The patients, or their family, gave their consent for the use of their personal and medical information for the publication of this case report.

## Surgical technique

**Fig. 2:**
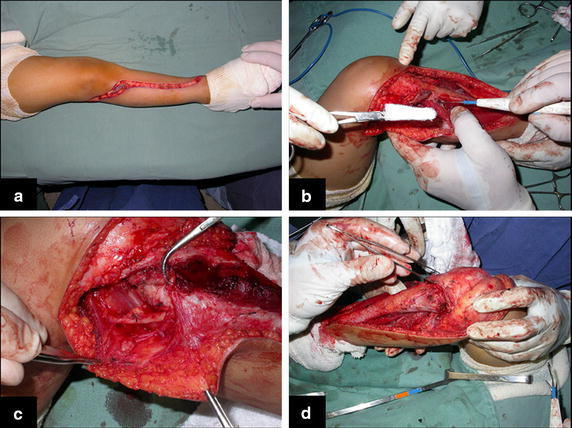
**a** Single incision, **b** opening of the perimysium and lateralization of the anterior tibial muscle, **c** dissection of the proximal part of the fibula and **d** dissection of tibial epiphysis

With the patient on supine position, a single incision is used. Starting above the proximal tibiofibular joint, the incision bends to the anterior tibial crest and down along it, bending again medially a few inches below the previously planned fibular osteotomy (Fig. 2a). The anterior tibialis muscle is exposed. Its perimysium is opened and the muscle is retracted laterally, leaving the inner layer of the perimysium attached to the tibial periosteum, in order to preserve the wide margin of tumor resection (Fig. 2b). The neck of the fibula is identified and the common peroneal nerve is dissected. The proximal tibiofibular joint is addressed and the joint capsule, along with the anterior and posterior ligaments, popliteal ligament, fibular collateral ligament and the femoral biceps tendon are released from the fibular head (Fig. 2c).

**Fig. 3:**
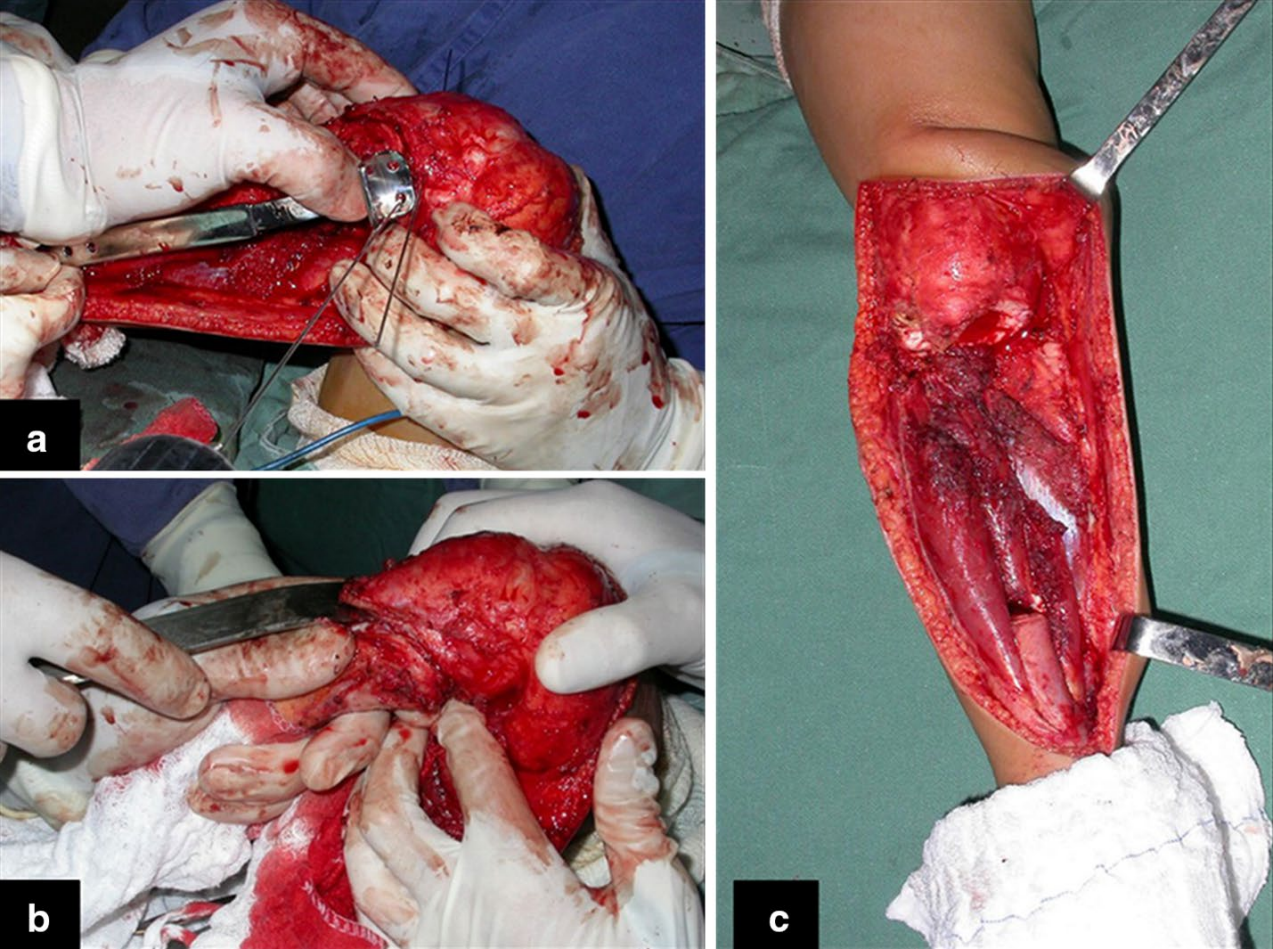
**a** Introduction of a wire-guide in the epiphysis and checking the position for the plate. **b** Separation of the tibial epiphysis from the tumor by transepiphyseal osteotomy and **c** tumor resected

The proximal epiphysis of the tibia and the anterior tuberosity are isolated from the metaphyseal region (Fig. 2d). A Kirschner wire is inserted horizontally trough the epiphysis where the proximal fixation of the plate will take place. The position of the plate is checked in this moment (Fig. 3a). The tumoral bone segment to be resected is measured, and oncologic margins are added. The distal osteotomy of the tibia at the diaphyseal region is performed. The posterior muscles attached to this portion of the bone are detached proximally, leaving the epiphyseal region that will be separated from the tumor by transepiphyseal osteotomy, and preserving as much of the epiphyseal bone and articular cartilage as possible. The tumor is now completely dissected and removed (Fig. 3b, c).

**Fig. 4:**
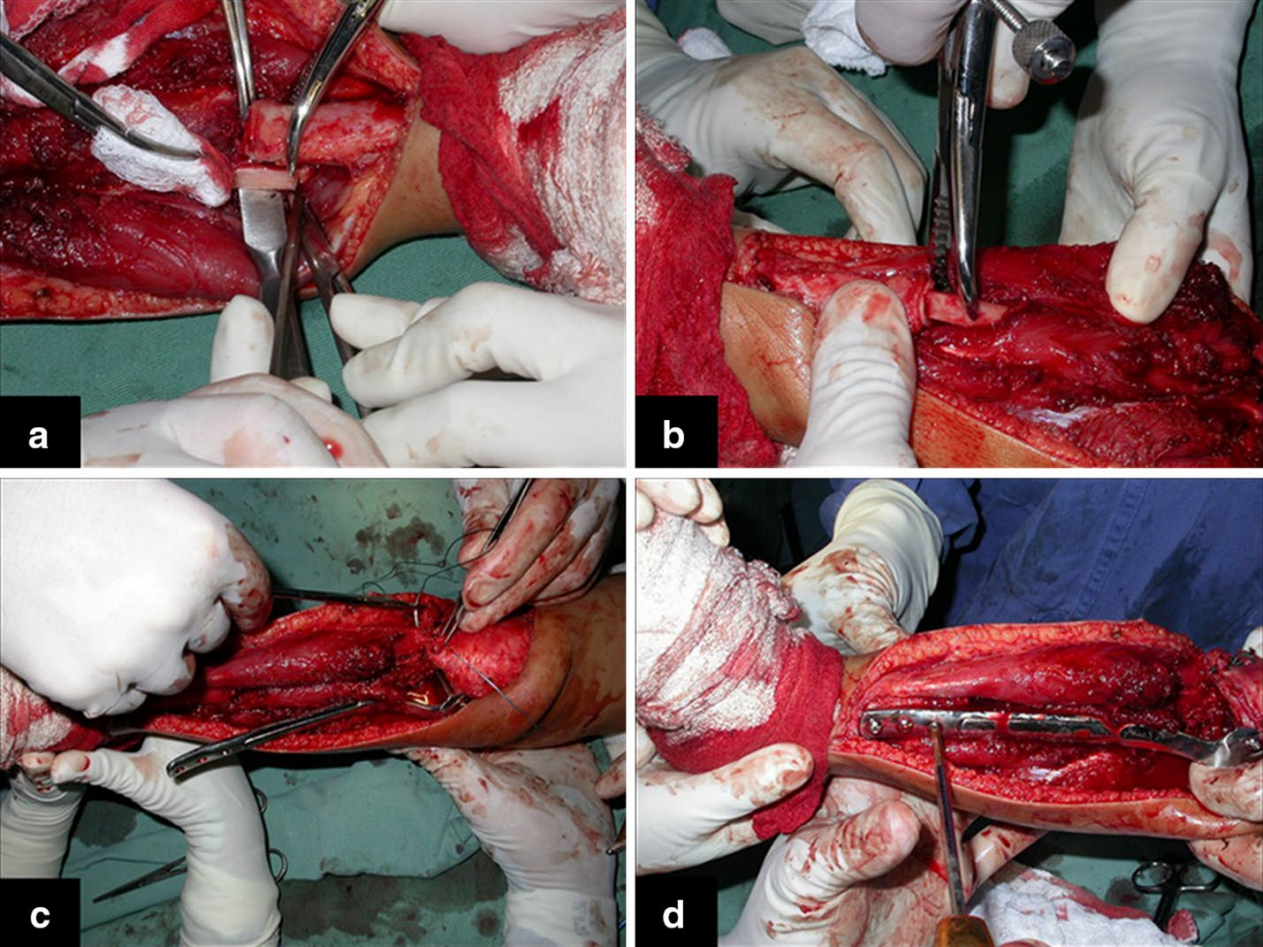
**a** Small periosteal removal from the fibula, **b** nailing the fibula in the medullary canal of the tibia, **c** repositioning of the fibula under the center of the tibial plateau and reinsertion of the lateral ligament and **d** proximal and distal osteosynthesis with screws

The bone gap is replaced by the ipsilateral fibula, which at this moment is isolated from the tibiofibular joint and the lateral collateral ligament. Two cm of periosteum is removed from the fibular shaft where the distal osteotomy will take place (Fig. 4a). After osteotomy, this segment of the fibular bone without its periosteum will be inserted in the bone marrow of the tibial shaft (Fig. 4b). The proximal segment of the fibula is medially transferred to the center of the remaining tibial epiphysis, along with all its muscles and nurturing arteries. The cartilage of the proximal epiphysis of the fibula is gently removed, so it can allow bone consolidation between the remaining proximal tibial epiphysis and the transposed fibula (Additional file 1: Video 5). The fibular collateral ligament is reinserted to the lateral periosteum of the tibia (Fig. 4c).

**Fig. 5:**
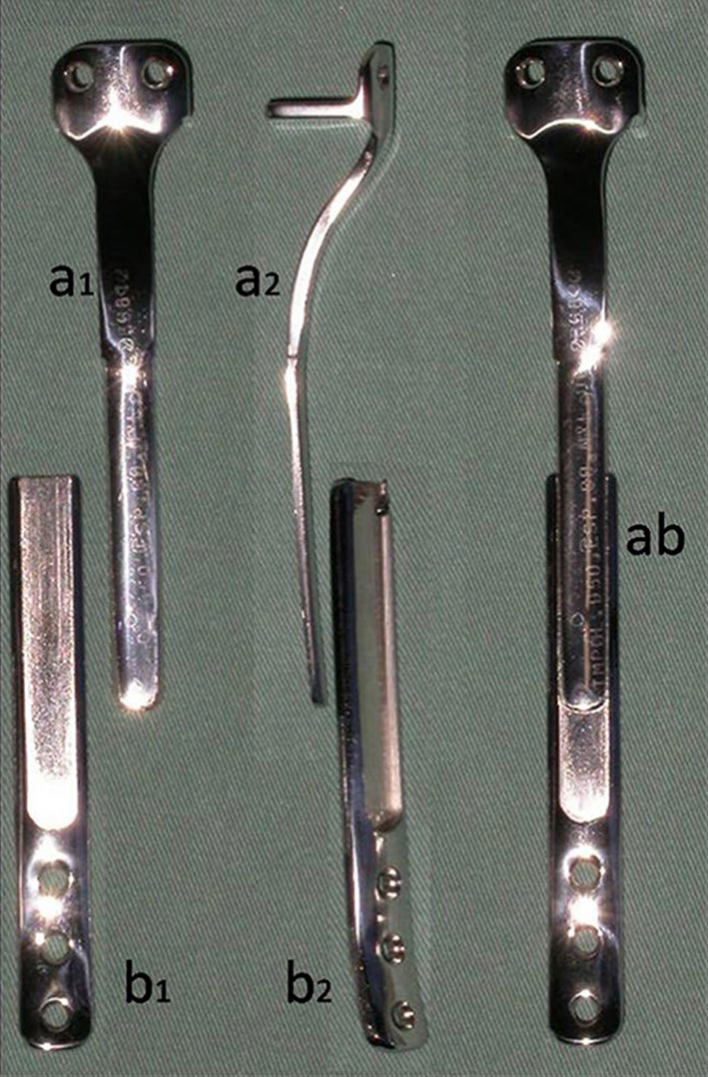
*a1* proximal plate, front view, *b1* distal plate, front view, *a2* proximal plate, side view, *b2* distal plate, side view and *ab* fitting of the two plates, mounting the device

The osteosynthesis with screws is performed and a Baptista’s extendable internal fixation device, previously tailored for each case (Baptista and Yonamine 2001), is placed on the medial side of the leg (Fig. 4d). This device, made in Brazil by IMPOL, consists of two plates connected by a trapezoidal shaped rail interlocking that allows longitudinal sliding between them, but creates stability in all other directions (Additional file 2: Video 1) (Fig. 5a1, b1). The proximal plate has a platform to support the remaining portion of the tibial plateau and screw holes for attachment to the epiphysis (Fig. 5a2). The distal plate is low profile, to facilitate its coverage by the skin of the medial leg, and has holes for the screws in the tibial diaphysis (Fig. 5b2). The channels on each plate fit each other, stabilizing the junction while allowing slippage (Fig. 5ab). This device allows lengthening according to the fíbula longitudinal growth. It also provide axial compression when weight bearing starts.

**Fig. 6:**
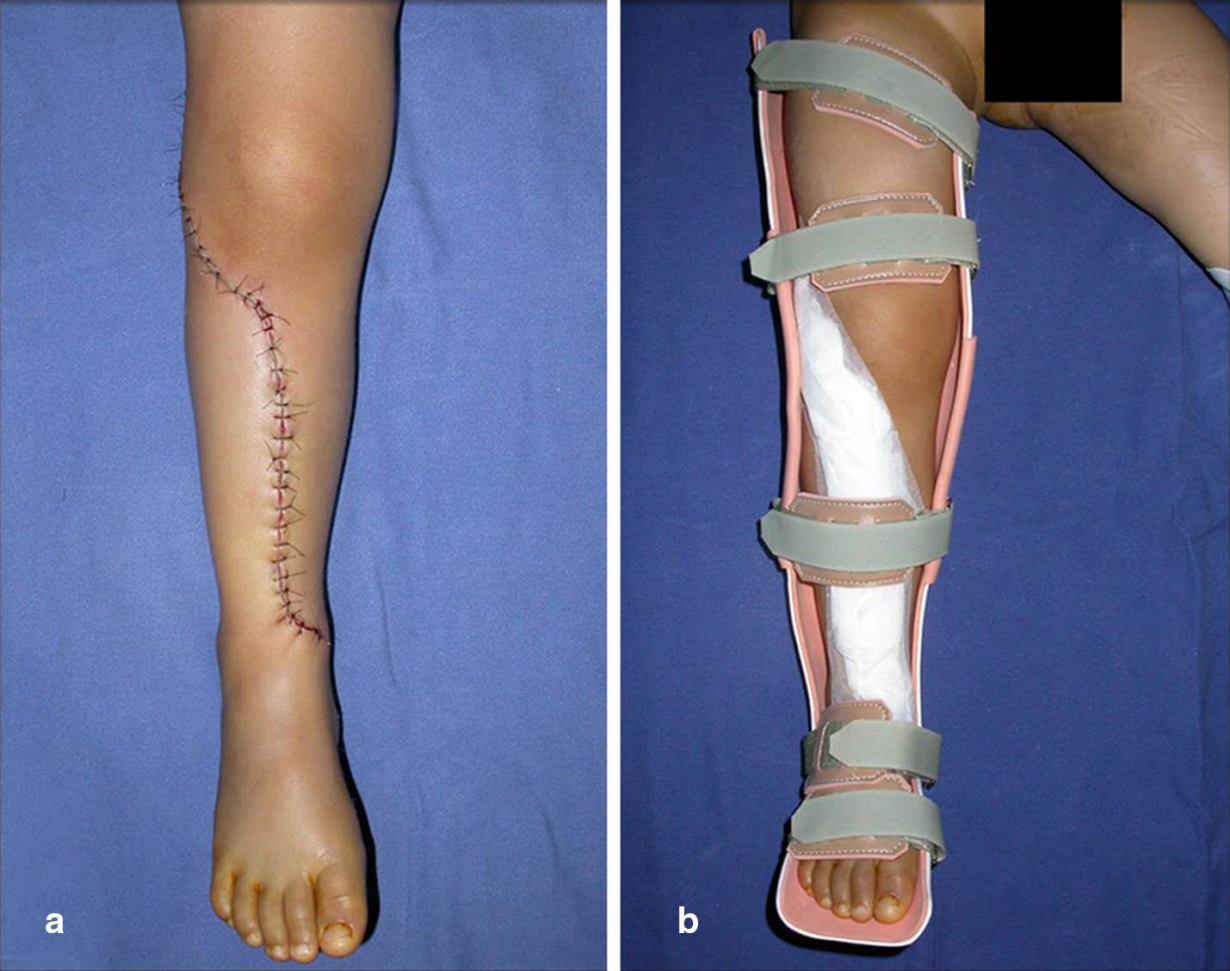
**a** Surgical wound and **b** custom made orthesis

The harvested fibula is interposed between the tibial epiphysis and the distal portion of the tibia. The surrounding soft tissues are reattached. After checking for vascular patency of the lateral side of the fibula, a closed vacuum wound drain is placed and the soft tissues are anatomically approximated. The limb is immobilized with an orthesis until osseous union of the proximal and distal junctions and hipertrophy of the fibula are radiographically confirmed (Additional file 3: Video 2) (Fig. 6), which usually occurs from 3 to 8 months postoperatively (Additional file 4: Video 3). Full weight bearing is authorized according to radiography consolidation and fibular hypertrophy (Additional file 5: Video 4).

## Case presentation

### Case 1

A 12 year old male patient with osteosarcoma of the proximal right tibia underwent wide tumor resection, with preservation of the proximal tibial epiphysis. The proximal fibula was medially transferred with its physis to the tibial epiphysis, preserving its blood supply, and osteosynthesis was performed with an extendable internal fixation device. After surgery, the limb was kept in an orthesis.

In the fourth postoperative month, radiographic evidence of consolidation was observed and load bearing was initiated with crutches. Full weight bearing started when fibular hypertrophy was radiographically evidenced, which occured at 14 months pos operatively. During follow up the patient returned to his full activities.

**Fig. 7:**
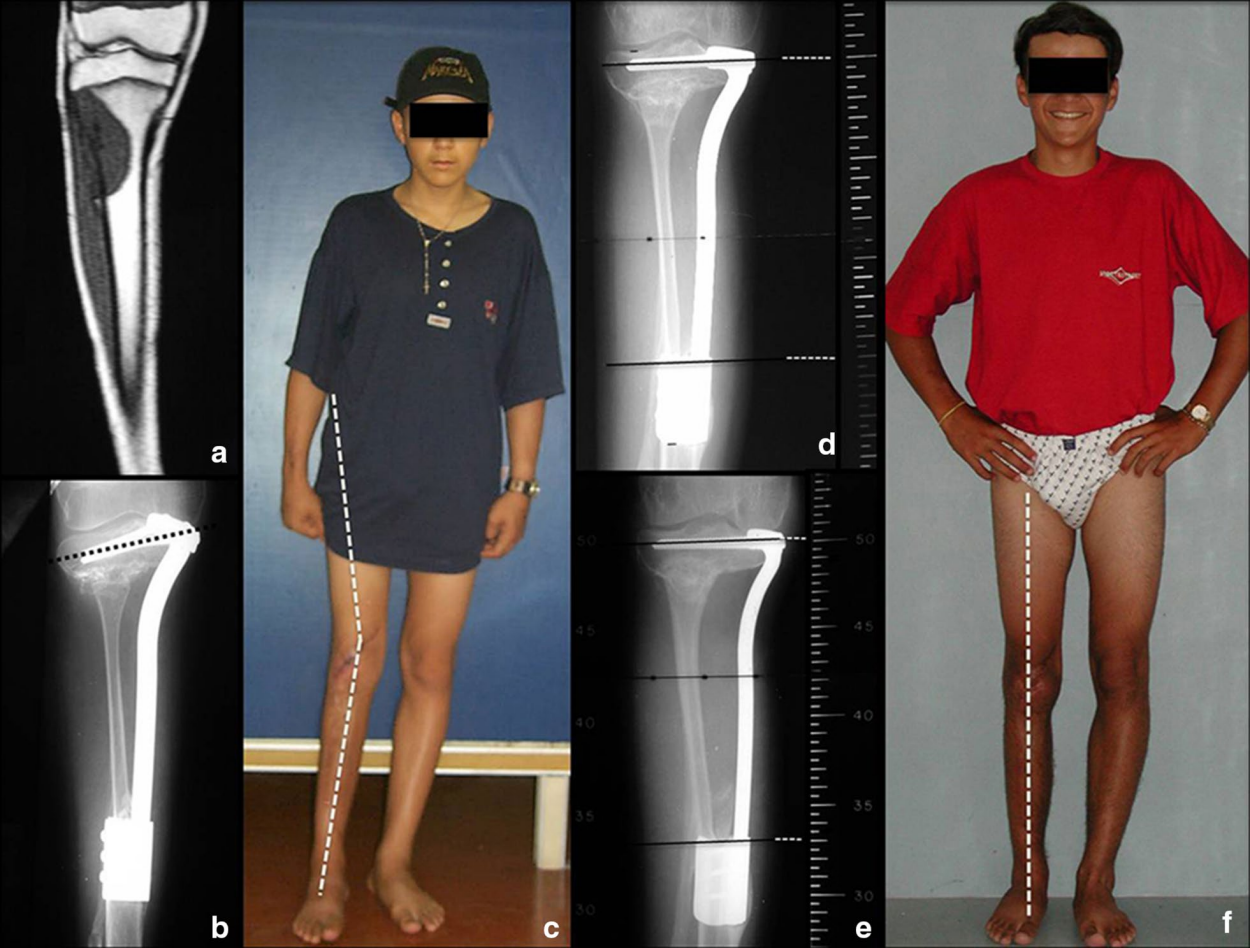
**a** Preoperative magnetic resonance, **b** 4 months postoperative radiograph showing the slope of the tibial epiphysis screws, **c** patient at 4 months after surgery with valgus knee deviation, **d** patient at 1 year and 2 months after surgery, with fixed valgus deformity and **e** 1 year and 2 months postoperative radiograph showing fibula hypertrophy, screws tilt correction and growth of 0.75 cm and **f** radiography postoperative 2 years and 2 months with growth of 1.2 cm

**Fig. 8:**
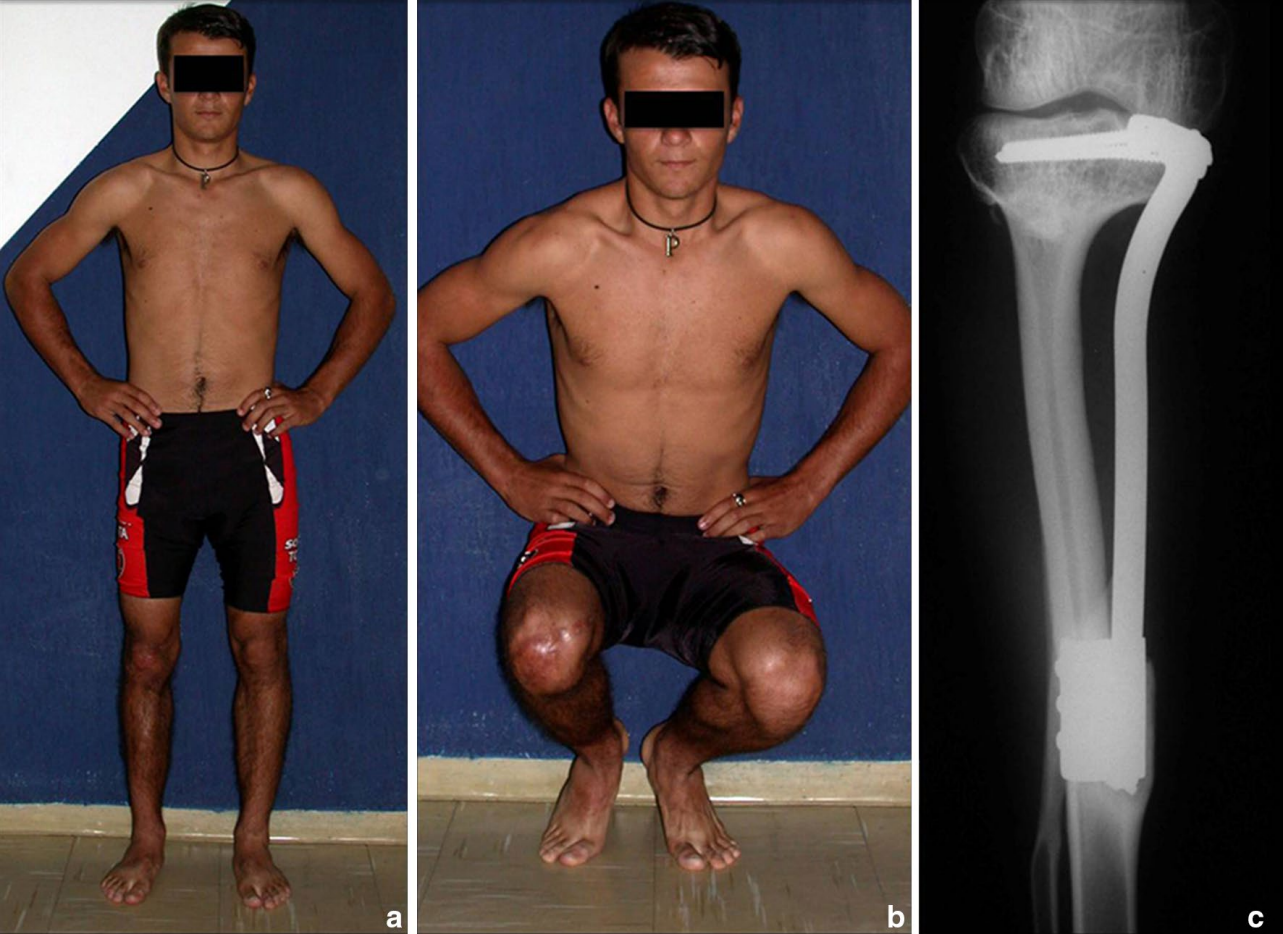
**a** patient 3 years and 7 months postoperatively, at full load, **b** 3 years, 7 months, bending under load and good function of the knee, and **c** radiograph 3 years, 7 months, fibula hypertrophied, already fully tibializated

In this case, we did not use fix angle screws in the proximal plate, which resulted in valgus deviation that was clinically observed and radiographically evidenced by tilting of the screws (Fig. 7a–c). The patient underwent the first scanometry of the lower limbs one year after surgery, when 0.75 cm fibular growth was observed and reorientation of the screws was done (Fig. 7b–e). Spontaneous correction of the angular deviation was clinically observed and flattening of the screws was radiographically documented, confirming the fibular longitudinal growth and the sliding of the device. The second scanometry, held 26 months postoperatively, demonstrated 1.2 cm growth of the transposed fibula (Fig. 7f). The patient is now a 26 year old man who has been followed up for 14 years without recurrences. He has equalized, satisfactory functioning lower limbs (Additional file 6: Video 6) (Fig. 8).

### Case 2

**Fig. 9:**
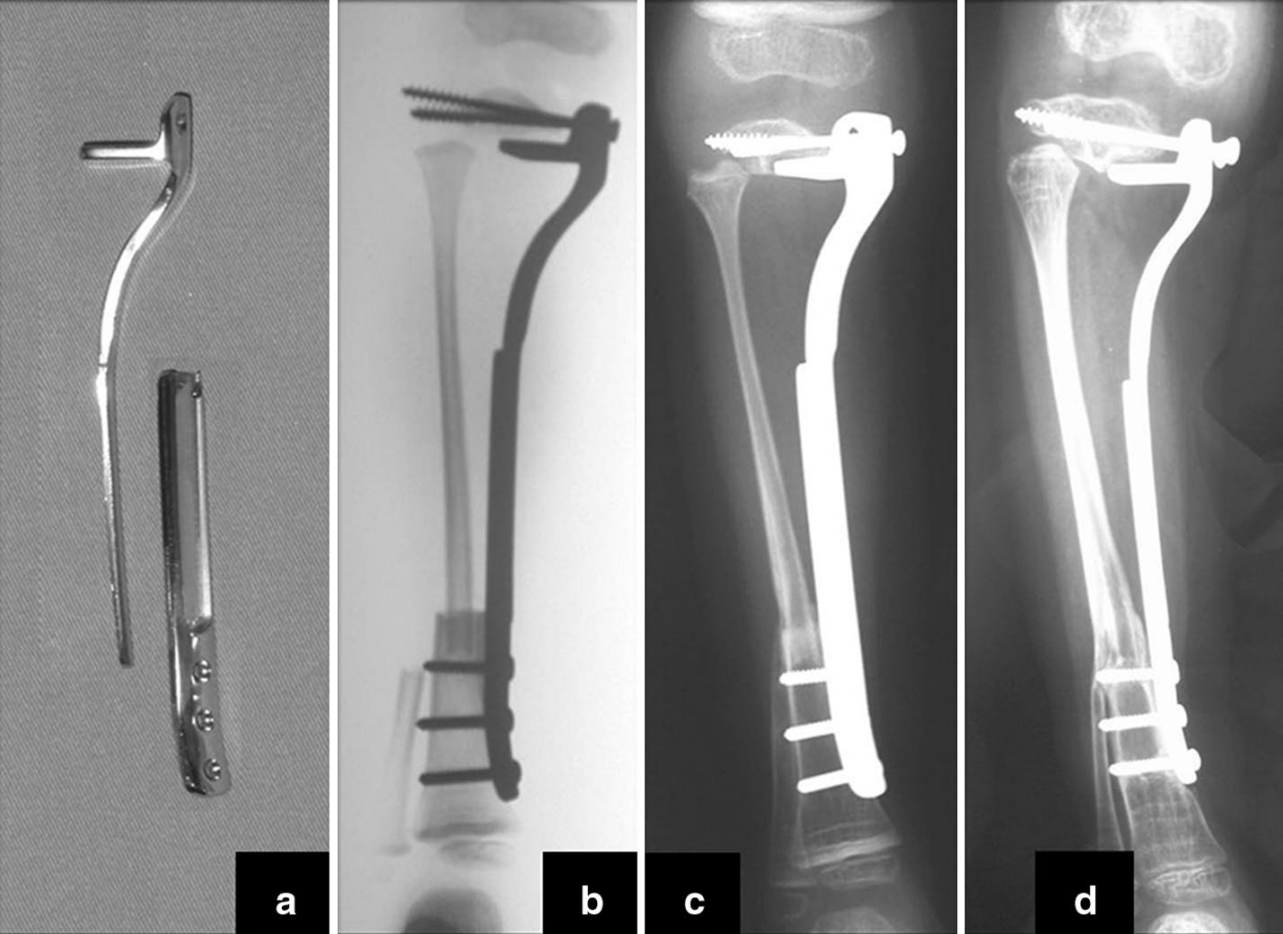
**a ** The proximal-plate with a horizontal support, for proximal tibia epiphisial support, **b** immediate postoperative radiograph, **c** 3 months postoperative radiograph, showing the distal tibio-fibular consolidation and **d** 6 months postoperative radiograph, showing hypertrophy the fibula

A 31 months old male patient presenting with Ewing’s sarcoma of the proximal right tibia underwent tumor resection with preservation of the proximal tibial epiphysis. The proximal fibula and its physis were medially transferred to the center of the tibial epiphysis, maintaining its blood supply. The osteosynthesis was performed using an extendable internal fixation device. In this case, the proximal plate was improved by creating a support to the remaining tibial plateau, aiming to improve stability and prevent angular deviations (Fig. 9b). Slots were made at every 3 mm of the distal plate to help observation of sliding between the plates, which would evidence fibular growth. After surgery, the limb was kept in an orthesis.

Load bearing was initiated in the third postoperative month. The patient continued on adjuvant chemotherapy and, in the fourth month, resumed walking without orthesis. During the first 8 months of follow up, approximately 0.3 cm growth of the transposed fibula was observed. Distal bone healing and initial fibular hypertrophy were radiographically confirmed (Fig. 9c, d). Non bone healing has occurred in the proximal fibula with the remnant epiphysis. Even though, full weight bearing occurred at 9 months after surgery (Additional file 5: Video 4), demonstrating the stability promoted by the Baptista’s extendable internal fixation device. After that period the patient died due to chemotherapy related complications.

## Discussion

Reconstruction of bone defects resulting from resection of tumors compromising the growth cartilage of the proximal tibia in children represents a challenge to the orthopedist. Due to the low frequency of these lesions, this is a rare situation, in which our technique is for a specific indication: Proximal tíbia tumors resections when the growth line must be removed for oncological reason, but the epiphysis can be preserved, in children with remnant limb growth potential.

Yoshida et al. (2010), has compared multiple reconstruction alternatives at the knee level after tumor resection. They demonstrated that when epiphyseal maintenance is feasible, the vascularized fibular graft or callotasis techniques offers the highest score in the MSTS rating system (Enneking et al. 1993).

Distraction osteogenesis with external fixator eliminates the need of graft and allows padding of the bone defects created by tumor resection through callostasis. It requires, however, long periods with an external fixator device, which increases the risk of infection in patients potentially immunosuppressed by adjuvant oncological treatment (Kapukaya et al. 2000).

Bone grafts have been used for reconstruction of proximal tibial resections, especially in cases where the tibial epiphysis can be preserved (Honoki et al. 2008), which occurs in up to 20 % of cases (Norton et al. 1991; Simon and Bos 1980). When the graft is not vascularized, the technique presents high rates of fracture or non-union, and it does not prevent further limb discrepancy (Muscolo et al. 2004). Weitao et al. (2012) reported the use of allograft in the proximal tibia in 5 patients after tumor resection preserving the epiphysis. Delayed bone healing in Allograft-host junction were seen in all cases.

Transposition of the fibula shows advantages over the allograft in the bone healing process. Since it is a bone-muscle flap with natural vascularization, bone turnover is preserved and actively participates in the process of bone healing, while the growth potential of the physis is maintained. As seen in Figs. 7 and 9, the fibula undergoes progressive hypertrophy and strengthening, in contrast to allograft, which may fail even years after integration (Date et al. 1996; Hriscu et al. 2006).

The transfer of the fibula with its growth plate as a reconstruction method requires long term rehabilitation and late resuming of loadbearing ambulation, until bone healing of the tibia and the transposed fibula occurs. Hypertrophy of the fibula, which was reported in our two cases, evidences that the bone has achieved enough resistance required for loadbearing. Since this is a biological reconstruction method, once consolidation and hypertrophy occur, it can be considered a definitive solution, carried out with a single surgical procedure. The preservation of the proximal epiphysis of the tibia while maintaining the articular surface of the knee by trans epiphyseal osteotomy represents a mechanical advantage. It is also a necessary condition to use a fibular transposition. The maintenance of longitudinal growth of the transposed fibular segment, as documented in our two patients, is of major importance, since it can prevent or minimize the final discrepancy of the lower limbs length after growth.

Capanna et al. (2007) presented a original solution for long bones intercalary defects reconstruction associating massive allograft with vascularized fibular autograft. Good results were presented in 57 patients with proximal tibia reconstruction, nevertheless this technique does not address final limb discrepancy. The authors developed a good alternative to reconstructions of bone defect of the proximal tibia, but require microsurgery technique, which increases the cost of the procedure and the availability of bone allograft compatible in size with the patient, which is not widely available in some countries.

Our technique has the advantage to waive any method of vascular reconstruction of the fibular segment, since vessels are preserved. Fibular hypertrophy and longitudinal growth of the transplanted bone segments were observed in the two reported cases. Distal bone healing was succeeded in both cases. Proximally, there was a non union in the second case, due to the non resection of the proximal fibular articular cartilage which is essential for bone healing. This patient was schedule to a second procedure to resect the articular cartilage of the proximal fibula, but he had a clinical complication, from chemotherapy, which led to death. During the first 8 months after surgery, however, growth of at least 0.3 cm of the transposed fibula was documented, suggesting preservation of the growth potential (Fig. 9).

The first patient developed a valgus deformity of the right knee in the immediate postoperative period, which was corrected within the first post-operative year, after bone growth and correction of slope of the screws (Fig. 7). Long term resolution of the tibial reconstruction defect was observed in this patient, as documented by the equal length and normal function of lower limbs in adulthood. In spite of the restricted indication, we believe that our technique can positively affect the long term outcome of young patients undergoing reconstruction of proximal tibial defects.

Reconstruction of the proximal tibia with a megaprosthesis represents a therapeutic option when epiphysis must be resected. It allows limb preservation and early ambulation but High rates of complications have been reported such as infection, aseptic loosening, mechanical failure, and limitation to physical activities (Saghieg et al. 2010; Gosheger et al. 2006; Campanacci et al. 2010; Fang et al. 2013). This method requires multiple surgical procedures for revisions, reaching 42 % implant failures in 10 years follow up (Jeys et al. 2008). When epiphyseal maintenance is feasible, megaprosthesis has fewer indications. When early ambulation is priority for low expectation of life patients.

## Conclusions

The authors believe the excellent longterm results in the first case and the ability to restore limb growth potential in both cases, avoiding further salvage surgical procedures, justify the application of this meticulous technique. The extensible internal fixation device stabilizes the reconstruction with the ipsilateral fibula and allows bone growth through sliding of the plates.

The purpose technique is indicated in intercalary proximal tibial resection, when the growth plate is removed for oncological purpose, in young children. Especially with high growth potential. The technique is not indicated in cases of low expectation of life due to the time required to initiate ambulation. In these situations a megaprosthesis might be preferred. More patients should be included on future studies to validate the reproducibility of this new technique.

## Additional files

10.1186/s40064-016-2042-7 Cartilage removal from the proximal epiphysis of the fibula, allow bone consolidation between the remaining proximal tibial epiphysis and the transposed fibula.
10.1186/s40064-016-2042-7 Internal sliding fixation device, demonstrating the extendable length.
10.1186/s40064-016-2042-7 Patient 2—two weeks postoperatively, removing homemade splint. Demonstrating stable knee.
10.1186/s40064-016-2042-7 Patient 2—two months postoperatively, demonstrating good, non painful, knee function.
10.1186/s40064-016-2042-7 Patient 2—eight months postoperatively, ambulating without crutches assistance, no pain, and good speed.
10.1186/s40064-016-2042-7 Patient 1—ten years and eight months postoperatively. Ambulating without crutches assistance, no pain, inferior limbs equalized in length and with great function.
